# Phylum Rotifera in Peru: a review of studies on biodiversity and annotated checklist of taxa

**DOI:** 10.3897/zookeys.1277.156127

**Published:** 2026-04-09

**Authors:** Elian Rojas-Baez, Iris Samanez, Luz Marina Soto, María José Pardo, Maciej Karpowicz, Evangelia Michaloudi, Georgia Stamou, Patricio R. de Los Ríos-Escalante, Carlos López

**Affiliations:** 1 Departamento de Limnología, Museo de Historia Natural, Universidad Nacional Mayor de San Marcos, Av. Arenales 1256, Jesús María, Lima, Peru Universidad Nacional Mayor de San Marcos Lima Peru; 2 Carrera de Ingeniería Ambiental, Facultad de Ciencias Agrícolas, Universidad Agraria del Ecuador, Guayaquil, Ecuador Universidad Agraria del Ecuador Guayaquil Ecuador; 3 Laboratorio de Ecología de Sistemas Acuáticos (Plancton), Centro de Ecología y Evolución, Instituto de Zoología y Ecología Tropical, Universidad Central de Venezuela, Caracas 1053, Venezuela Universidad Central de Venezuela Caracas Venezuela; 4 Department of Hydrobiology, Faculty of Biology, University of Bialystok, Bialystok, Poland University of Bialystok Bialystok Poland; 5 Department of Zoology, School of Biology, Aristotle University of Thessaloniki, 54124 Thessaloniki, Greece Aristotle University of Thessaloniki Thessaloniki Greece; 6 Department of Biological and Chemical Sciences, Faculty of Natural Resources, Catholic University of Temuco, Temuco, Chile Department of Biological and Chemical Sciences, Faculty of Natural Resources, Catholic University of Temuco Temuco Chile; 7 Nucleus of Environmental Sciences, Faculty of Natural Resources, Catholic University of Temuco, Temuco, Chile Nucleus of Environmental Sciences, Faculty of Natural Resources, Catholic University of Temuco Temuco Chile; 8 Escuela Superior Politécnica del Litoral, (ESPOL), Centro de Agua y Desarrollo Sustentable, Campus Gustavo Galindo, Guayaquil, Ecuador Escuela Superior Politécnica del Litoral, (ESPOL), Centro de Agua y Desarrollo Sustentable, Campus Gustavo Galindo Guayaquil Ecuador; 9 Departamento de Biología, Facultad Experimental de Ciencias, Universidad del Zulia, Maracaibo, Venezuela Universidad del Zulia Maracaibo Venezuela

**Keywords:** Neotropics, Peruvian Amazonia, rotifers, South America, Tropical Andes, zooplankton diversity

## Abstract

Peru is a megadiverse country, home to some of the world’s most important biodiversity hotspots. This paper systematically reviews published studies on the phylum Rotifera in Peru, aiming to identify key features, knowledge gaps, and research challenges, thereby contributing to the conservation of rotifers. A brief history of rotifer studies is presented, and the first annotated checklist is provided of all rotifer taxa recorded according to their most recent taxonomic status, valid nomenclature, and department distribution. The number of studies on Peruvian rotifer fauna is limited, with only 35 publications. These papers are primarily in the Spanish language and are published in national journals. Ecological studies, often conducted on a short-term basis, frequently dominate classical taxonomic works. Additionally, many departments and types of freshwater environments in the country remain understudied. To date, 203 valid rotifer species have been recorded. This number is unrepresentative, as more than half of the departments lack documented records. Most recorded rotifer species in Peru are from freshwater habitats in the eastern of the country, particularly near the Amazon Basin. Several important gaps are highlighted, including the need for precise revision of existing records, promoting taxonomic harmonization, and enhancing sampling efforts.

## Introduction

The phylum Rotifera, commonly known as rotifers, comprises a group of small organisms (body size 50–2,000 μm) and includes approximately 2,030 species distributed worldwide (Segers 2007; Wallace and Snell 2010). Rotifers possess various mechanisms, including the capacity to enter a dormant or resting phase, which enables survival and resistance to environmental conditions in diverse aquatic (mostly freshwater) or limnoterrestrial habitats (macrophytes, psammon, mosses, lichens, and phytotelmata) (Fontaneto and De Smet 2015). They play a crucial role in the aquatic food web (Wallace and Snell 2010; Gilbert 2022), and several species are widely used as model organisms and biological tools (Declerck and Papakostas 2017; Serra and Fontaneto 2017; Rico-Martínez et al. 2017; Won et al. 2017; Hagiwara and Marcial 2019; Gribble 2021; Shaleh et al. 2024).

Like other microscopic organisms that historically have been understudied (Vicente 2010; Anthony et al. 2023), the biodiversity of rotifer species across many regions worldwide remains largely unrecognized (Fontaneto et al. 2006; Segers 2007, 2008). Particularly in the Neotropics, the enormous environmental heterogeneity has facilitated Rotifera to become one of the most speciose groups of inland waters zooplankton (Andrade-Sossa et al. 2023; Castilho-Noll et al. 2023; Duré et al. 2023; González et al. 2023; López et al. 2023). However, our knowledge about Neotropical rotifers is still dispersed and limited, especially concerning the tropical Andes region and eastern Amazon, where many threats promote habitat destruction and biodiversity losses (Asner et al. 2017; Sánchez-Cuervo et al. 2020; Campero et al. 2024). Recent studies from these countries have begun to establish a solid foundation for advancing rotifer research through reviews, updated checklists, and species distribution records (López et al. 2020; Andrade-Sossa et al. 2023; Elmoor-Loureiro et al. 2023; González et al. 2023; López et al. 2023; Fernández et al. 2024).

Peru is one of the 17 megadiverse countries (Fajardo et al. 2014) and covers two important biodiversity zones: the Tropical Andes and the Tumbes-Chocó-Magdalena hotspot (Noss 1990; Myers 1988). Peru is also characterized by extreme topographic complexity, with the arid Pacific coast, the high Andes, and the Amazon basin, as well as marked climatic diversity ranging from tropical to temperate to arid conditions (Myers 1988). The National System of Natural Protected Areas by the Peruvian State represents ~15.2% of the country, to conserve a representative sample of its biodiversity. Nevertheless, important gaps in conservation coverage have been identified, so a review and expansion of the system is required (Fajardo et al. 2014). Priority areas for conservation are primarily determined based on expert criteria, with emphasis on regions of diversity and endemism (Rodríguez and Young 2000), as has been documented for certain organism groups and marine biodiversity (Angulo 2009; Fernandez-Baca et al. 2007). However, as in many other countries, freshwater biodiversity — particularly that of small organisms, such as protozoa, rotifers, and microcrustaceans, which constitute the core of freshwater zooplankton — remains largely unknown and underestimated. Consequently, these groups are rarely considered in research and conservation policies (Cuesta et al. 2017; Oliveira et al. 2017; Bax and Francesconi 2019; Mendoza-Ponce et al. 2020; Kleemann et al. 2022).

Here, we conducted a systematic literature review to analyze studies published on Peruvian rotifers, identifying their main features. We also provide a brief historical overview and present the first annotated checklist of all rotifer taxa recorded in Peru, based on their most recent taxonomic status, valid nomenclature, and distribution. This work aims to identify knowledge gaps and research challenges and to contribute to the conservation of their biodiversity.

## Methods

The present work is based on a systematic review of scientific publications reporting rotifer species in Peru. A literature search was conducted using keywords “Rotifer,” “Rotifers,” “Rotifera” and “Peru” in both English and Spanish across databases including Web of Science, Zoological Record, Scopus, Scielo and Google Scholar. The digital search was supplemented with scientific articles in printed form (old papers, not digitalized) and the articles cited in reviewed articles (Duré et al. 2023).

Only studies on rotifers done inside the Peruvian territory and/or using rotifer species or strains originating from these were considered for analysis. Only 35 publications were considered for baseline data analysis. Based on this baseline data (Suppl. material 1), a brief history of studies and a bibliometric analysis were conducted. A graphical representation of publications over the years provides evidence of the time trends. We categorized the publications according to the language used, the institutions involved (national, international, or both), and the publication type (journal papers, book chapters, and books) using titles and abstracts of the publications. Each publication was assigned to only one category. Additionally, based on the review of the publication texts, we categorized the topics (field ecology, experimental ecology, or taxonomy), the administrative divisions (departments), the types of environments studied (e.g., lakes, lagoons, ponds, reservoirs, wetlands, rivers, and other artificial habitats), and whether the studies focused on the entire zooplankton assemblage or exclusively on rotifers. For the ecological studies, features such as the ecological level (individual, population, community) and duration: short (< 5 years) or long-term (> 5 years), were highlighted according to Castilho-Noll et al. (2023). All graphics were generated using the “ggplot2” package in R v. 4.4.1.

For the checklist, we reviewed the full text of all papers, including those from non-indexed journals, to exclude genus-level identifications. Reports from studies with a doubtful rotifer species identification process, involving species classified as species inquirenda, absence of information on the department or place of origin were not considered in the elaboration of the annotated checklist (See Suppl. material 1). The taxonomic status and ranges of rotifer species were identified using the Rotifer World Catalog (Jersabek and Leitner 2013). Species names, authorship, synonyms, and spellings were verified and updated according to the recent List of Available Names (**LAN**) (Jersabek et al. 2018a, 2018b; Segers et al. 2012). The records were assigned based on the political territorial division of Peru at the department level, considering the Constitutional province of Callao as a department. An overview of the number of rotifer species recorded across different departments is illustrated in a map created using QGIS software v. 3.28.1. Species with distribution and reported for the Neotropical region were analyzed using information from the Rotifer World Catalog (**RWC**) (Jersabek and Leitner 2013) and Segers (2007).

## Results

### Brief history

The study of rotifers in Peru dates back to the mid-19^th^ century, beginning with the description of *Polyarthra hexaptera* Schmarda, 1859; currently a species inquirenda (Jersabek et al. 2018b) reported by Schmarda (1859) after a global expedition. Subsequent reports on rotifer species were part of invertebrate surveys conducted in the Andean departments of southwestern Peru, particularly in and around Lake Titicaca (Murray and Wailes 1913; De Beauchamp 1939; Thomasson 1956; Richerson et al. 1977). A re-evaluation of some of these previous studies reporting *Notholca foliacea* (Ehrenberg, 1838) (Murray and Wailes 1913; De Beauchamp, 1939) was conducted based on additional literature (Hollowday and Hussey 1989). Subsequently, several studies described the rotifer diversity in freshwater habitats from the Peruvian Amazon and central high Andean lakes (Koste 1988; Samanez 1988, 1991; Zambrano and Burger 1992; Samanez and Riofrío 1995; Samanez and Zambrano 1995).

During the past two decades, rotifers reports have frequently been incorporated in limnological characterizations of inland waterbodies (Riofrío et al. 2003; Ortega et al. 2006), and ecological studies of zooplankton communities (Iannacone and Alvariño 2006; Iannacone et al. 2013a; Chávez-Veintemilla et al. 2020; Flores 2022), with some studies primarily focusing on wetlands within the central coastal desert (Iannacone and Alvariño 2007a, b; Paredes et al. 2007; Iannacone et al. 2013b; Toscano and Severino 2013; Cepeda et al. 2018). Other research has employed advanced techniques to examine the resistance and revival of rotifers from extreme environments in the southern Peruvian Andes (Schmidt et al. 2017), and a recent study has further enhanced our understanding of the rotifer community in this region, offering new records and insights for the fauna of Peru (Karpowicz et al. 2025).

Additionally, research on native rotifer strains has explored their potential as bioindicators (Alayo and Iannacone 2002; Osorio et al. 2022) and their applications in local aquaculture (Alayo and Iannacone 2001; Cisneros 2011; Murrieta-Morey et al. 2015; Huanacuni-Pilco and Espinoza-Ramos 2019; Aguilar-Samanamud et al. 2022; Ismiño-Orbe et al. 2022). Moreover, the only study conducted at the national level that employed an integrative approach—combining molecular, morphological, and physiological aspects— was focused on the *Brachionus plicatilis* Müller, 1786 species complex from desert environments in Peru, leading to the identification of new subspecies, *B. paranguensis ventanillensis* and *B. koreanus santodomingensis*, both described by Sánchez-Dávila et al. (2021).

### Bibliometric analysis

**Figure 1.**
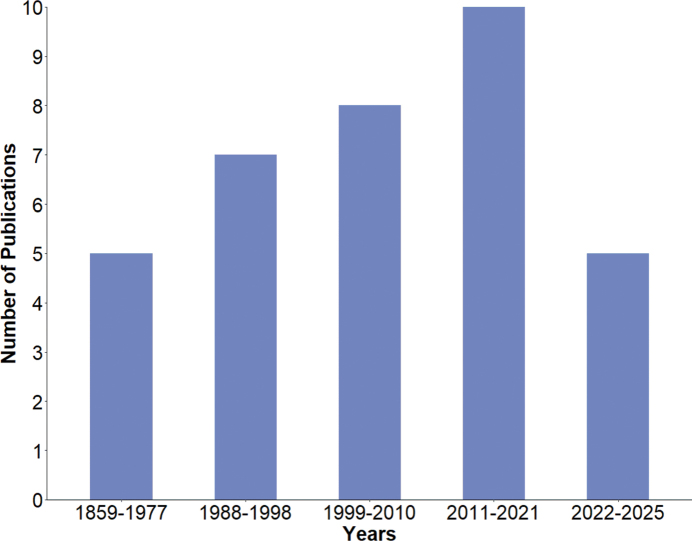
Temporal trend of the number of publications concerning rotifer studies in Peru.

The bibliometric analysis focused on 35 publications (Suppl. material 1). The graphical representation of publications over time covers the period from 1859 to 2025. The earliest records dates back to 1859, but between that year and 1977, only five publications appeared, indicating very sporadic research activity. From the mid-20^th^ century until the late 1980s and early 1990s, the mean frequency of publications was approximately one every 20 years. After this period, the frequency increased to an average of one publication every four years. For the last two decades, a clear upward trend is evident, with publications appearing roughly every two years since 2013. Thus, a marked growth in rotifer-related research has been observed in recent decades (Fig. 1).

**Figure 2.**
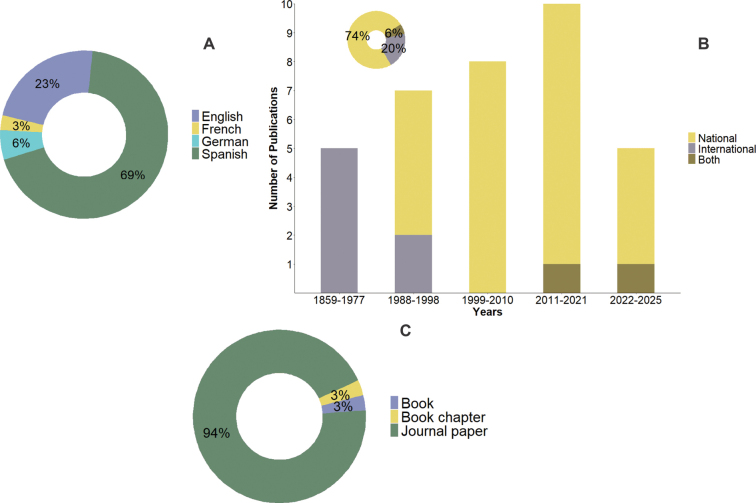
Publications by A. Language; B. Institutional category; C. Type of publication on rotifers from Peru.

Most publications were written in Spanish, with fewer written in English, and a few papers in German and French (Fig. 2A). Most of this literature has been authored by researchers affiliated with Peruvian institutions, whose contributions first emerged in the late 1980s and have shown an increasing trend in recent years. In contrast, international researchers represent a small proportion and were the primary contributors until the late 1970s. During the last decade, only two studies have involved collaboration between Peruvian and international researchers (Fig. 2B). Most of the publications appeared in national journals, with one book and a single book chapter comprising the remainder (Fig. 2C).

The primary topics addressed in publications, in descending order of representation were field ecology (19), experimental ecology (9), and taxonomy (7). Most of these studies were based on samplings from natural inland water bodies, such as lakes, ponds, and rivers, while wetlands, reservoirs, and lagoons were mentioned less frequently. Additionally, various types of artificial environments, including fish farms, water intakes, water channels, aquaculture hatcheries, and cattle watering holes, were also referenced. No publications on limnoterrestrial habitats were found.

The review covers 12 department regions of Peru, with Lima and Puno standing out due to having the highest number of publications, nine and seven, respectively. These are followed by departments with two to five publications, such as Loreto, Ucayali, Huánuco, Junín, Callao, and Cusco. In contrast, regions like Ica, Madre de Dios, Tacna, and Piura are represented by only one publication each. Overall, 14 publications focused on lowland systems indicating modest predominance of research in Amazonian and coastal waterbodies, while 12 addressed highland systems, showing that Andean lakes and rivers also received considerable attention.

**Figure 3.**
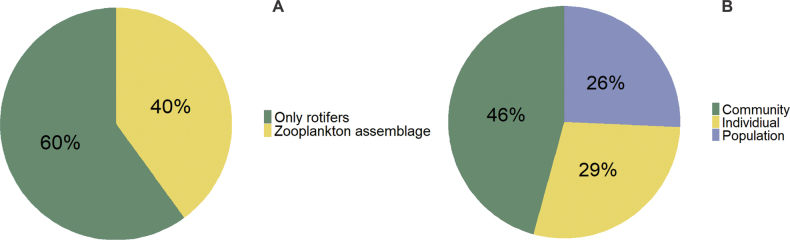
A. Primary group; B. Organization level focusing in publications on rotifers from Peru.

Based on the ecological scope of each publication, only a few studies have focused on a single rotifer species (Fig. 3A). In contrast, the majority have examined entire zooplankton communities, including rotifers as one component. A smaller proportion of studies have specifically targeted the entire rotifer communities (Fig. 3B). Additionally, it is important to highlight that all studies were short-term duration.

### Checklist of species

**Figure 4.**
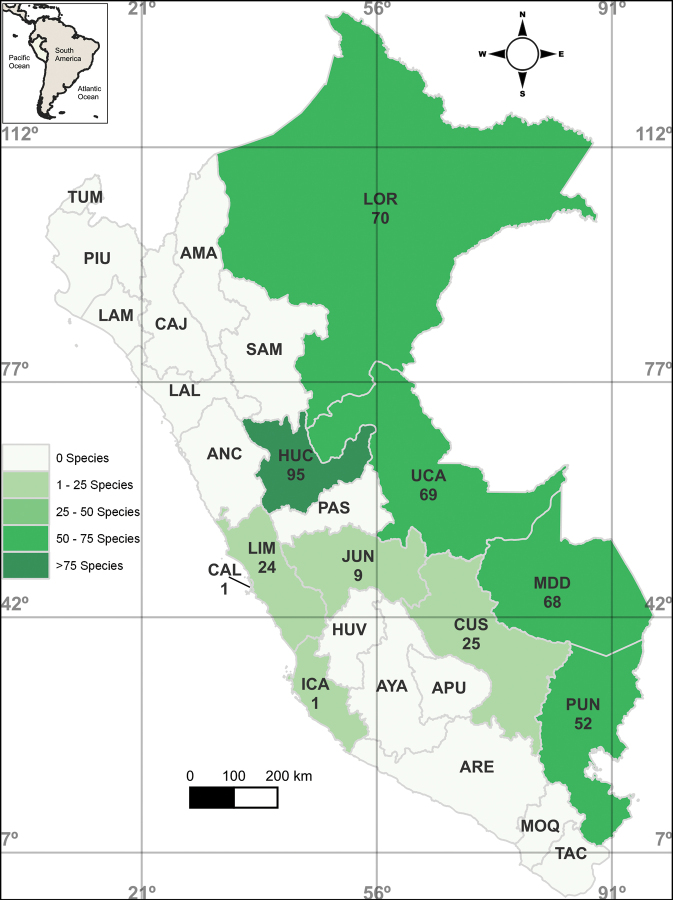
Locations and number of rotifer species recorded from each department of Peru. **AMA** Amazonas **ANC** Ancash **APU** Apurímac **ARE** Arequipa **AYA** Ayacucho **CAJ** Cajamarca **CAL** Callao **CUS** Cusco **HUV** Huancavelica **HUC** Huánuco **ICA** Ica, **JUN** Junín **LAL** La Libertad **LAM** Lambayeque **LIM** Lima **LOR** Loreto **MDD** Madre de Dios **MOQ** Moquegua **PAS** Pasco **PIU** Piura **PUN** Puno **SAM** San Martin **TAC** Tacna **TUM** Tumbes **UCA** Ucayali.

The annotated checklist of species, based on the literature search (Suppl. material 1), resulted in 512 records of rotifers in Peru, corresponding to 203 valid species (187 monogononts and 16 bdelloids) (Table 1, Suppl. material 2). All recorded taxa were reported from inland water bodies (Fig. 4). Most species exhibit a worldwide distribution, while only 13 (6%) have a Neotropical distribution (Table 1). No endemic or invasive species in Peru were identified.

**Table 1.** List of rotifer species recorded in Peru. For abbreviations, see Fig. 4.

Species	Department
**Subclass Bdelloidea Hudson, 1884**	
**Order Philodinida Melone & Ricci, 1995**	
**Family Habrotrochidae**	
*Habrotrocha angusticollis* (Murray, 1905)	PUN, HUC
**Family Philodinidae Ehrenberg, 1838**	
*Ceratotrocha cornigera* (Bryce, 1893)	PUN
*Dissotrocha aculeata* (Ehrenberg, 1830)	HUC, LOR, UCA, MDD
*Dissotrocha macrostyla* (Ehrenberg, 1838)	HUC
*Macrotrachela habita* (Bryce, 1894)	CUS
*Macrotrachela multispinosa* Thompson, 1892	CUS
*Macrotrachela musculosa* Milne, 1886	CUS
*Macrotrachela papillosa* Thompson, 1892	CUS, PUN
*Macrotrachela plicata* (Bryce, 1892)	PUN
*Philodina acuticornis* Murray, 1902	PUN, JUN, LIM
*Philodina megalotrocha* Ehrenberg, 1832	HUC
*Pleuretra triangularis* Murray, 1913 *	PUN
*Rotaria neptunia* (Ehrenberg, 1830)	HUC, LOR, UCA, MDD
*Rotaria rotatoria* (Pallas, 1766)	PUN, HUC
*Rotaria tardigrada* (Ehrenberg, 1830)	HUC
*Rotaria tridens* (Montet, 1915)	HUC
**Subclass Monogononta Plate, 1889**	
**Order Collothecaceae Harring, 1913**	
**Family Collothecidae Harring, 1913**	
*Collotheca campanulata* (Dobie, 1849)	HUC
*Collotheca ornata* (Ehrenberg, 1830)	PUN, HUC
**Order Flosculariaceae Harring, 1913**	
**Family Conochilidae Harring, 1913**	
*Conochilus dossuarius* Hudson, 1885	HUC
*Conochilus unicornis* Rousselet, 1892	PUN
**Family Flosculariidae Ehrenberg, 1838**	
*Beauchampia crucigere* (Dutrochet, 1812)	HUC
*Floscularia ringens* (Linnæus, 1758)	HUC
*Limnias ceratophylli* Schrank, 1803	HUC
*Limnias melicerta* Weisse, 1848	HUC
*Ptygura beauchampi* Edmondson, 1940	HUC
*Ptygura crystallina* (Ehrenberg, 1834)	HUC
*Ptygura longicornis* (Davis, 1867)	LIM
*Ptygura melicerta* Ehrenberg, 1832	HUC
*Ptygura wilsonii* (Anderson & Shephard, 1892)	UCA, LOR
*Sinantherina ariprepes* Edmondson, 1939	LOR
*Sinantherina semibullata* (Thorpe, 1889)	LOR, MDD
*Sinantherina spinosa* (Thorpe, 1893)	LOR
**Family Hexarthridae Bartoš, 1959**	
*Hexarthra bulgarica* (Wiszniewski, 1933)	LIM
*Hexarthra fennica* (Levander, 1892)	PUN
*Hexarthra intermedia* (Wiszniewski, 1929)	LOR, MDD
*Hexarthra mira* (Hudson, 1871)	UCA
**Family Testudinellidae Harring, 1913**	
*Pompholyx complanata* Gosse, 1851	PUN
*Pompholyx sulcata* Hudson, 1885	PUN
*Testudinella ahlstromi* Hauer, 1956	UCA
*Testudinella incisa* (Ternetz, 1892)	HUC
*Testudinella mucronata* (Gosse, 1886)	PUN, UCA
*Testudinella mucronata haueriensis* Gillard, 1967 *	HUC, UCA, LOR, MDD
*Testudinella ohlei* Koste, 1972 *	LOR, UCA, MDD
*Testudinella patina* (Hermann, 1783)	PUN, HUC, UCA, MDD, LOR
**Family Trochosphaeridae Harring, 1913**	
*Filinia longiseta* (Ehrenberg, 1834)	HUC, UCA, LOR, LIM
*Filinia opoliensis* (Zacharias, 1898)	UCA, LOR, MDD
*Filinia pejleri* Hutchinson, 1964	UCA, LOR, MDD
**Order Ploima Hudson & Gosse, 1886**	
**Family Asplanchnidae Eckstein, 1883**	
*Asplanchna girodi* Guerne, 1888	CUS
*Asplanchna priodonta* Gosse, 1850	LOR, UCA, MDD, LIM
*Asplanchna silvestrii* Daday, 1902	PUN
*Asplanchnopus multiceps* (Schrank, 1793)	PUN
**Family Brachionidae Ehrenberg, 1838**	
*Anuraeopsis fissa* (Gosse, 1851)	HUC, LOR, MDD
*Anuraeopsis navicula* Rousselet, 1911	MDD, UCA
*Brachionus ahlstromi* Lindeman, 1939	UCA
*Brachionus angularis* Gosse, 1851	LOR, LIM
*Brachionus austrogenitus* Ahlstrom, 1940 *	LOR
*Brachionus bennini* Leissling, 1924	UCA, LOR, MDD
*Brachionus bidentatus* Anderson, 1889	PUN
*Brachionus calyciflorus* sl Pallas, 1766	PUN, UCA, LOR, LIM
*Brachionus caudatus* Barrois & Daday, 1894	UCA, LOR
*Brachionus dimidiatus* Bryce, 1931	JUN, LIM
*Brachionus dolabratus* Harring, 1914 *	UCA, LOR, MDD
*Brachionus falcatus* Zacharias, 1898	UCA, LOR, MDD
*Brachionus gessneri* Hauer, 1956 *	LOR
*Brachionus havanaensis* Rousselet, 1911	LOR
*Brachionus ibericus* Ciros-Pérez, Gómez & Serra, 2001	LIM
*Brachionus koreanus santodomingensis* Sanchez-Davila & al., 2021	ICA
*Brachionus mirabilis* Daday, 1897	HUC, LOR, UCA, MDD
*Brachionus mirus* Daday, 1905 *	UCA, LOR, MDD
*Brachionus paranguensis ventanillensis* Sanchez-Davila & al., 2021	CAL
*Brachionus plicatilis* sl Müller, 1786	LIM
*Brachionus quadridentatus* Hermann, 1783	PUN, HUC, UCA, LOR, MDD, LIM
*Brachionus urceolaris* Müller, 1773	PUN, LIM
*Brachionus variabilis* Hempel, 1896	PUN
*Brachionus zahniseri* Ahlstrom, 1934	UCA, LOR, MDD
*Keratella americana* Carlin, 1943	UCA, LOR, MDD
*Keratella cochlearis* (Gosse, 1851)	CUS, HUC, UCA, LOR, MDD, JUN, LIM, PUN
*Keratella lenzi* Hauer, 1953	UCA, LOR, MDD
*Keratella quadrata* (Müller, 1786)	CUS, LOR, PUN,
*Keratella tecta* (Gosse, 1851)	CUS, PUN
*Keratella testudo* (Ehrenberg, 1832)	LIM
*Keratella tropica* (Apstein, 1907)	PUN, HUC, LOR, MDD, LIM
*Keratella valga* (Ehrenberg, 1834)	MDD
*Notholca striata* (Müller, 1786)	LOR
*Notholca walterkostei* José de Paggi, 1982	PUN, CUS
*Plationus patulus* (Müller,1786)	HUC, UCA, LOR, MDD
*Plationus patulus macracanthus* (Daday, 1905)	HUC, UCA, LOR, MDD
*Platyias leloupi* Gillard, 1957	LOR, UCA, MDD
*Platyias quadricornis* (Enhrenberg, 1832)	HUC, UCA, MDD, LOR, JUN
**Family Dicranophoridae Harring, 1913**	
*Dicranophoroides caudatus* (Ehrenberg, 1834)	HUC
*Dicranophorus epicharis* Harring & Myers, 1928	HUC
*Dicranophorus forcipatus* (Müller, 1786)	HUC
*Encentrum saundersiae* (Hudson, 1885)	PUN
**Family Epiphanidae Harring, 1913**	
*Epiphanes clavulata* (Ehrenberg, 1831)	HUC
*Epiphanes macroura* (Barrois & Daday, 1894)	UCA, LOR, HUC, MDD, LIM
*Epiphanes senta* (Müller, 1773)	PUN, LIM
*Mikrocodides chlaena* (Gosse, 1886)	HUC
**Family Euchlanidae Ehrenberg, 1838**	
*Beauchampiella eudactylota* (Gosse, 1886)	HUC, UCA, MDD
*Dipleuchlanis propatula* (Gosse, 1886)	HUC, UCA, MDD, LOR,
*Euchlanis dilatata* (Ehrenberg, 1832)	UCA, HUC, LOR, MDD, LIM, PUN
*Euchlanis incisa* Carlin, 1939	HUC
*Euchlanis lyra* Hudson, 1886	HUC
*Euchlanis parva* Rousselet, 1892	PUN
*Euchlanis pyriformis* Gosse, 1851	PUN
*Euchlanis semicarinata* Segers, 1993	CUS
**Family Gastropodidae Harring, 1913**	
*Ascomorpha saltans* Bartsch, 1870	PUN
**Family Ituridae Sudzuki, 1964**	
*Itura aurita* (Ehrenberg, 1830)	HUC
**Family Lecanidae Remane, 1933**	
*Lecane aculeata* (Jakubski, 1912)	HUC
*Lecane amazonica* (Murray, 1913) *	HUC, LOR, MDD
*Lecane bulla* (Gosse, 1851)	CUS, HUC, LOR, MDD, UCA, PUN
*Lecane closterocerca* (Schmarda, 1859)	CUS, HUC, PUN
*Lecane cornuta* (Müller, 1786)	HUC, UCA, LOR, MDD
*Lecane crepida* Harring, 1914	UCA, MDD
*Lecane curvicornis* (Murray, 1913)	HUC, LOR, MDD, UCA
*Lecane doryssa* Harring, 1914	UCA
*Lecane elsa* Hauer, 1931	LOR, UCA, MDD
*Lecane ercodes* Harring, 1914	UCA, HUC, MDD
*Lecane furcata* (Murray, 1913)	HUC, MDD
*Lecane haliclysta* Harring & Myers, 1926	MDD
*Lecane hamata* (Stokes, 1896)	CUS, HUC, UCA, LOR, MDD
*Lecane inopinata* Harring & Myers, 1926	HUC
*Lecane leontina* (Turner, 1892)	UCA, LOR, MDD, JUN, LIM
*Lecane levistyla* (Olofsson, 1917)	UCA
*Lecane ludwigii* (Eckstein, 1883)	LOR, UCA, MDD
*Lecane luna* (Müller, 1776)	CUS, HUC, LIM, PUN
*Lecane lunaris* (Ehrenberg, 1832)	CUS, PUN, LOR, HUC, MDD, JUN, LIM
*Lecane marchantaria* Koste & Robertson, 1983 *	HUC
*Lecane monostyla* (Daday, 1897)	UCA, MDD, JUN, LIM
*Lecane nana* (Murray, 1913)	PUN
*Lecane nodosa* Hauer, 1938	UCA
*Lecane opias* (Harring & Myers, 1926)	PUN
*Lecane papuana* (Murray, 1913)	HUC, UCA, LOR, MDD
*Lecane pertica* Harring & Myers, 1926	LOR, MDD
*Lecane proiecta* Hauer, 1956 *	LOR, UCA, MDD
*Lecane pumila* Rousselet, 1906	PUN
*Lecane pyriformis* (Daday, 1905)	CUS, PUN, HUC
*Lecane quadridentata* (Ehrenberg, 1830)	HUC, UCA, LOR, MDD
*Lecane remanei* Hauer, 1964 *	MDD
*Lecane rugosa* (Harring, 1914) *	UCA
*Lecane stenroosi* (Meissner, 1908)	LOR, MDD
*Lecane stichaea* Harring, 1913	HUC
*Lecane styrax* (Harring & Myers, 1926)	UCA
*Lecane wulferti* Hauer, 1956	HUC
**Family Lepadellidae Harring, 1913**	
*Colurella adriatica* Ehrenberg, 1831	UCA, LOR, MDD, PUN
*Colurella obtusa* (Gosse, 1886)	LOR, MDD
*Colurella uncinata* (Müller, 1773)	CUS, HUC, LIM, PUN
*Colurella uncinata bicuspidata* (Ehrenberg, 1830)	PUN
*Lepadella acuminata* (Ehrenberg, 1834)	HUC
*Lepadella amphitropis* Harring, 1916	UCA, HUC
*Lepadella costata* Wulfert, 1940	LOR, HUC
*Lepadella donneri* Koste, 1972 *	HUC, UCA, MDD
*Lepadella elongata* Koste, 1992	PUN
*Lepadella latusinus* (Hilgendorf, 1899)	LOR
*Lepadella ovalis* (Müller, 1786)	CUS, HUC, MDD, UCA, LOR
*Lepadella patella* (Müller, 1773)	CUS, PUN, HUC, MDD, UCA, LIM
*Lepadella patella oblonga* (Ehrenberg, 1834)	UCA
*Lepadella quadricarinata* (Stenroos, 1898)	UCA, MDD
*Lepadella rhomboides* (Gosse, 1886)	CUS, HUC
**Family Lindiidae Harring & Myers, 1924**	
*Lindia torulosa* Dujardin, 1841	PUN
**Family Mytilinidae Harring, 1913**	
*Mytilina bisulcata* (Lucks, 1912)	LOR
*Mytilina michelangellii* Reid & Turner, 1988	MDD
*Mytilina mucronata spinigera* (Ehrenberg, 1830)	LOR
*Mytilina trigona* (Gosse, 1851)	MDD
*Mytilina unguipes* (Lucks, 1912)	LOR, MDD
*Mytilina ventralis* (Ehrenberg, 1830)	HUC, LOR, UCA, MDD
**Family Notommatidae Hudson & Gosse, 1886**	
*Cephalodella boettgeri* Koste, 1988	HUC
*Cephalodella catellina* (Müller, 1786)	PUN
*Cephalodella forficula* (Ehrenberg, 1830)	HUC, PUN
*Cephalodella gibba* (Ehrenberg, 1830)	CUS, PUN, HUC
*Cephalodella gigantea* Remane, 1933	HUC
*Cephalodella gracilis* (Ehrenberg, 1830)	PUN, HUC
*Cephalodella hollowdayi* Koste, 1986 *	HUC, LOR, UCA, MDD
*Cephalodella mucronata* Myers, 1924	HUC
*Cephalodella panarista* Myers, 1924	HUC
*Enteroplea lacustris* Ehrenberg, 1830	HUC
*Monommata actices* Myers, 1930	UCA
*Monommata grandis* Tessin, 1890	HUC
*Monommata maculata* Harring & Myers, 1930	HUC
*Notommata aurita* (Müller, 1786)	HUC
*Notommata collaris* Ehrenberg, 1832	HUC
*Notommata copeus* Ehrenberg, 1834	HUC
*Notommata glyphura* Wulfert, 1935	HUC
*Notommata voigti* Donner, 1949	CUS
*Resticula melandoca* (Gosse, 1887)	HUC
**Family Proalidae Harring & Myers, 1924**	
*Proales decipiens* (Ehrenberg, 1830)	HUC
*Proales theodora* (Gosse, 1887)	CUS
**Family Scaridiidae Manfredi, 1927**	
*Scaridium longicauda* (Müller, 1786)	HUC
**Family Synchaetidae Hudson & Gosse, 1886**	
*Polyarthra dolichoptera* Idelson, 1925	PUN
*Polyarthra remata* Skorikov, 1896	HUC
*Polyarthra vulgaris* Carlin, 1943	PUN, LOR, HUC, MDD, UCA
**Family Trichocercidae Harring, 1913**	
*Trichocerca bicristata* (Gosse, 1887)	HUC, UCA, LOR, MDD, JUN
*Trichocerca braziliensis* (Murray, 1913)	HUC
*Trichocerca chattoni* (Beauchamp, 1907)	LOR, HUC, UCA, MDD
*Trichocerca dixonnuttalli* (Jennings, 1903)	HUC
*Trichocerca mucosa* (Stokes, 1896)	UCA
*Trichocerca rattus* (Müller, 1776)	PUN
*Trichocerca similis* (Wierzejski, 1893)	HUC
*Trichocerca similis grandis* Hauer, 1965	UCA, LOR, MDD
*Trichocerca tenuior* (Gosse, 1886)	HUC
*Trichocerca tigris* (Müller, 1786)	HUC, MDD
**Family Trichotriidae Harring, 1913**	
*Macrochaetus sericus* (Thorpe, 1893)	LOR, UCA, MDD
*Trichotria tetractis* (Ehrenberg, 1830)	LOR, HUC, UCA
*Trichotria pocillum* (Müller, 1776)	CUS

* Species with only Neotropical distribution.

Nearly half of the total recorded species for the country come from the department of Huánuco (47%), followed by significant proportions from Ucayali (34%), Loreto (35%), Madre de Dios (34%), and Puno (26%). In contrast, other departments show notably fewer records, such as Lima (12%), Cusco (12%), Junín (4%), and Callao and Ica (1%) (Fig. 4).

Rotifer species from Peru are represented in 25 families and 52 genera. Most rotifer species belong to the families Brachionidae (6 genera with 38 species, 19%), Lecanidae (1 genera with 36 species, 18%) and Notommatidae (5 genera with 19 species, 9%). The most diverse and frequently reported genera are *Lecane* (36 species) and *Brachionus* (22 species) (Table 1).

## Discussion

The number of publications on Peruvian rotifers is limited, with only 35 studies available. However, not all of these were useful for compiling a reliable checklist of taxa (as can be seen in Suppl. material 1), as some only provided general ecological information without precise species identification.

First referenced data dates back to the 19^th^ century, while classical descriptive studies emerged in the 20^th^ century with significant advancements from the 1980’s decade until the present, a pattern frequently observed in rotifer and zooplankton research across other countries in the Neotropical region (Aoyagui and Bonecker 2004; González et al. 2023; Castilho-Noll et al. 2023; Fernández et al. 2024). Short-term ecological studies predominate the literature on Peruvian rotifers, overshadowing classical taxonomic research. Although many natural freshwater ecosystems remain understudied, the number of environments reviewed encompasses all aquatic habitats listed in the Peruvian national ecosystem catalog (MINAM, 2019) and documents rotifer diversity in less-studied areas, such as bogs, vernal pools, and ponds. Exploring these ecosystems can reveal a “hidden” diversity, as their structural complexity strongly shapes rotifer assemblages (Ejsmont-Karabin and Karpowicz 2021). Karpowicz et al. (2025) exemplified this by reporting the first Neotropical records of *Notommata voigti* Donner, 1949 and *Macrotrachela musculosa* Milne, 1886 (based on Segers, 2007) from subhabitats not previously considered in the Andean region, demonstrating that Peruvian rotifer fauna remains underrepresented even in relatively well-studied areas.

Most recorded rotifer species in Peru are from freshwater habitats in the eastern regions, particularly near the Amazon Basin, a primary hotspot of the country’s biological diversity (Rodríguez and Young 2000). This concentration of records underscores the uneven distribution of available data across the country. Moreover, research on Peruvian rotifer fauna is further constrained by the limited number of specialists in this taxon, which has not only reduced the development of taxonomic studies over time but has also shifted research efforts towards short-term ecological studies. Also, although only a few regions have contributed a comparatively higher number of publications (See Suppl. material 2), most records derive from studies conducted by internationally recognized rotifer specialists (e.g., Koste 1988) and national zooplankton researchers (e.g., Samanez 1988, 1991). This imbalance highlights how the geographic distribution of taxonomy experts constrains the understanding of the rotifer diversity in any country (Fontaneto et al. 2012; Ejsmont-Karabin 2019; López et al. 2025).

The recorded rotifer diversity in Peru represents a small proportion (8%) of all rotifer taxa recorded worldwide (Segers 2007, 2008). Nevertheless, the number of rotifer species is unrepresentative, as over half of the departments lack documented records (Fig. 4). In comparison to the rotifer diversity reported in South America, the number of recorded species from Peru is higher than in countries with smaller territorial areas, such as Uruguay (40 species) (Carballo et al. 2023), Chile (121 species) (De los Ríos-Escalante and Woelfl 2023) and Paraguay (71 species) (Duré et al. 2023). However, it is lower than in other neighboring countries, including Brazil (630 species) (Elmoor-Loureiro et al. 2023), Ecuador (287 species) (López et al. 2020), Colombia (253 species) (Andrade-Sossa et al. 2023) and Bolivia (279 species) (Fernández et al. 2024).

Although our review found no records of invasive species in Peru, some taxa, such as *Kellicottia bostoniensis* (Rousselet, 1908) and *Synchaeta jollyae* (Shiel & Koste, 1993) have already been documented in South America (Negreiros et al. 2012; Oliveira et al. 2019; Gomes et al. 2022; Villabona-González and López-Cardona 2022). These records may represent the initial stage of an invasion process and future occurrences in Peru are likely, given the connectivity of many aquatic environments to the Amazon Basin, which has been proposed as a potential dispersal pathway (Gomes et al. 2022), together with the broad adaptations and environmental tolerance of both species in the Neotropics (Negreiros et al. 2012; Gomes et al. 2022).

Among the neotropical rotifers described for the first time in Peru, *Cephalodella boettgeri* Koste, 1988 and *Pleuretra triangularis* Murray, 1913 have also been recorded in other regions and nearby countries (Krau 1967; Segers and Sanoamuang 2007). Similarly, Sánchez-Dávila et al. (2021) reported one *Brachionus* clade shared by populations from coastal South America, and another cosmopolitan lacking clear phylogeographic relationship. Together, these findings demonstrate that the taxa originally described from Peru do not represent endemism and highlight the need for future systematic studies to uncover biogeographical patterns, particularly in cryptic species (Bickford et al. 2007).

Although research on other inland water communities, such as fish and macroinvertebrates, is more advanced (Ortega et al. 2012; Arana-Maestre et al. 2021), studies on Peruvian freshwater zooplankton are scarce. This study, together with the cladoceran list compiled by Valdivia-Villar (1988), constitutes one of the few faunistic reviews devoted to this group. This highlights that research in this area remains limited when compared with the more extensive studies conducted in other Neotropical countries (López et al. 2023).

Additionally, although rotifers are often overlooked in biodiversity assessments because their wide dispersal and apparent cosmopolitanism make them seem less informative as biogeographic indicators (Segers 2008), the data presented here provide valuable insights into their distribution in Peru. This contributes to addressing a critical challenge in biological conservation for this country, such as correcting the overestimation of species numbers (Rodríguez and Young 2000; Bax and Francesconi 2019).

Since the regional review by Aoyagui and Bonecker (2004), knowledge of the rotifer fauna in Peru has been poorly documented. Thus, the present paper provides a valuable contribution to understanding Peruvian Rotifera diversity and lays the foundation for future research efforts.

Consequently, based on the available information, the most critical gaps that need to be addressed are as follows: (i) Precise taxonomic revision of existing records, supported by modern molecular tools, given the frequency of cryptic species in rotifers (Papakostas et al. 2016; Walczyńska et al. 2024). (ii) Closer collaboration with leading international taxonomists, together with increased research efforts at national and regional levels, is needed to promote taxonomic harmonization and generate additional benefits through scientific collaboration (Duan 2011; Cheng et al. 2020). (iii) More extensive sampling is required in both well-studied and underexplored regions, employing methodologies for characterizing active (planktonic) and latent forms of rotifers in sediments (Jeppesen et al. 2011).

### Recommendations

1. Expand research coverage: Broaden sampling efforts in unexplored Andean, coastal, and Amazonian regions, as well as in rivers, wetlands, limno-terrestrial habitats, and on host-associated rotifers, to reduce pronounced geographical and habitat biases.
2. Strengthen taxonomy: Apply integrative taxonomy to revise existing records and uncover cryptic species, while ensuring alignment with global databases. Special attention should also be given to the largely neglected Bdelloid rotifers.
3. Foster collaboration and capacity-building: Create strong national and regional research networks to counteract the shortage of taxonomists and ensure the formation of new generations of specialists. Such initiatives will also facilitate publishing in international journals, thereby increasing the global visibility and impact of Peruvian Rotifera research.

## Additional information

### Use of AI

No use of AI was reported.

### Author ORCIDs

Elian Rojas-Baez https://orcid.org/0000-0002-8222-2031

Iris Samanez https://orcid.org/0000-0001-8315-5339

Luz Marina Soto https://orcid.org/0000-0001-6996-3866

María José Pardo https://orcid.org/0000-0001-8829-4573

Maciej Karpowicz https://orcid.org/0000-0003-0711-5903

Evangelia Michaloudi https://orcid.org/0000-0001-8194-2865

Georgia Stamou https://orcid.org/0000-0002-0709-3698

Patricio R. de Los Ríos-Escalante https://orcid.org/0000-0001-5056-7003

Carlos López https://orcid.org/0000-0001-5267-7257

### Data availability

All of the data that support the findings of this study are available in the main text or Supplementary Information.

## Supplementary materials

Supplementary material 1 List of works resulting from the literature search Data type docx https://binary.pensoft.net/file/1589256This dataset is made available under the Open Database License (http://opendatacommons.org/licenses/odbl/1.0/). The Open Database License (ODbL) is a license agreement intended to allow users to freely share, modify, and use this Dataset while maintaining this same freedom for others, provided that the original source and author(s) are credited. Elian Rojas-Baez, Iris Samanez, Luz Marina Soto, María José Pardo, Maciej Karpowicz, Evangelia Michaloudi, Georgia Stamou, Patricio R. de Los Ríos-Escalante, Carlos López

Supplementary material 2 Checklist database Data type xlsx https://binary.pensoft.net/file/1589257This dataset is made available under the Open Database License (http://opendatacommons.org/licenses/odbl/1.0/). The Open Database License (ODbL) is a license agreement intended to allow users to freely share, modify, and use this Dataset while maintaining this same freedom for others, provided that the original source and author(s) are credited. Elian Rojas-Baez, Iris Samanez, Luz Marina Soto, María José Pardo, Maciej Karpowicz, Evangelia Michaloudi, Georgia Stamou, Patricio R. de Los Ríos-Escalante, Carlos López
