# Supplementary material for: Phylum Rotifera in Peru: a review of studies on biodiversity and annotated checklist of taxa
Source: Zookeys. 2026 Apr 9;1277:79–100. doi: 10.3897/zookeys.1277.156127 (PMC13087659; doi:10.3897/zookeys.1277.156127)
Supplement: Supplementary material 1 — List of works resulting from the literature search [file zookeys-1277-079_article-156127__-s001.docx]

**Supplementary Material 1.** List of works resulting from the literature search

(*) Studies excluded for the preparation of the annotated checklist of rotifer species due to one or more of the following reasons: reports involving species classified as species inquirenda, insufficient identification at the species level, or absence of information on the department or place of origin.

* Aguilar-Samanamud CP, Gaspar W, Inga G, Flores L, Sanchez P, Hernandez-Acevedo H, Borda-Soares R, Olivera-Galvez A (2022) Variables that intervene in the weight of rotifer biomass and fatty acids. Journal of the World Aquaculture Society 53(3): 741-753. <https://doi.org/10.1111/jwas.12848>

Alayo M, Iannacone J (2001) Crecimiento poblacional del rotífero eurihalino *Brachionus plicatilis hepatotomus* alimentado con la microalga *Nannochloris sp*. Boletín de Lima 123: 87–93.

* Alayo M, Iannacone J (2002) Ensayos ecotoxicológicos con petróleo crudo, diesel 2 y diesel 6 con dos subespecies de *Brachionus plicatilis* Müller 1786 (Rotifera: Monogononta). Gayana (Concepción) 66(1): 45–58.<http://dx.doi.org/10.4067/S0717-65382002000100007>

* Cepeda C, Iannacone J, Alvariño L (2018) Conexión trófico entre las comunidades planctónicas y la avifauna silvestre en Pantanos de Villa, Lima, Perú. Biotempo 15: 175–195.<https://doi.org/10.31381/biotempo.v15i2.2057>

Chávez-Veintemilla C, Pezo R, Vásquez E (2020) Diversidad Planctónica de los cuerpos de agua en la Reserva Nacional Allpahuayo Mishana, Loreto- Perú. Folia Amazónica 29: 353–370.<https://doi.org/10.24841/fa.v29i2.536>

Cisneros R (2011) Rendimiento poblacional del rotífero nativo *Brachionus* sp. “Cayman”, utilizando diferentes enriquecedores. Ecología Aplicada 10(1-2): 99–105.<https://doi.org/10.21704/rea.v10i1-2.419>

De Beauchamp P (1939) Rotifères et Turbellariés. Transactions of the Linnean Society of London 1: 51–79. <https://doi.org/10.1111/j.1096-3642.1939.tb00005.x>

Flores AJ (2022) Diversidad de zooplancton en el lago Titicaca durante la evaluación de biomasa de recursos pesqueros, Julio 2019. Informe IMARPE 49(4): 552–569.<https://repositorio.imarpe.gob.pe/handle/20.500.12958/5874>

Hollowday ED, Hussey CG (1989) A re-appraisal of two members of the genus *Notholca* from the Andes, with a note on the fine structure of the lorica of *N*. *foliacea* (Ehrenberg). Hydrobiologia 186: 319–324. <https://doi.org/10.1007/BF00048927>

* Huanacuni-Pilco JI, Espinoza-Ramos LA (2019) Producción de alimento vivo para la investigación en acuicultura de peces marinos en la UNJBG, Tacna. Ciencia & Desarrollo 17: 82–86.<https://doi.org/10.33326/26176033.2018.22.749>

Iannacone J, Alvariño L (2006) Diversidad del zooplancton en la reserva nacional de Junín, Perú. Ecología Aplicada 5: 175–181. [www.scielo.org.pe/scielo.php?script=sci_arttext&pid=S1726-22162006000100024&lng=es&nrm=iso&tlng=es](http://www.scielo.org.pe/scielo.php?script=sci_arttext&pid=S1726-22162006000100024&lng=es&nrm=iso&tlng=es)

Iannacone J, Alvariño L (2007a) Diversidad de invertebrados acuáticos de la bocatoma de la atarjea en el Río Rímac, Lima Perú durante 1999. Biotempo 7: 61–75.<https://doi.org/10.31381/biotempo.v7i0.874>

Iannacone J, Alvariño L (2007b) Diversidad y abundancia de comunidades zooplanctónicas litorales del humedal Pantanos de Villa, Lima, Perú. Gayana (Concepción) 71(1): 49–65.<https://doi.org/10.4067/S0717-65382007000100006>

Iannacone J, Salazar N, Alvariño L, Argota G (2013a) Rotifers and other littoral zooplankton species from the Andean lagoons of Paca and Ñahuinpuquio, Jauja, Junin, Peru. Neotropical Helminthology 7: 133–142.

Iannacone J, Alvariño L, Jimenez R, Argota G (2013b) Diversidad del plancton y macroozoobentos como indicador alternativo de calidad de agua del río Lurín en el distrito de Cieneguilla, Lima-Perú. The Biologist 11(1): 79–95. <https://doi.org/10.24039/rtb2013111432>

Ismiño-Orbe RA, Fernández-Méndez C, Ramírez-Arrarte P, Burga-Ríos J, Alván-Aguilar M (2022) Influencia de microalgas en el crecimiento del rotífero de agua dulce *Brachionus calyciflorus* (Pallas, 1766), Loreto, Perú. Folia Amazónica 31: 47–55. <https://doi.org/10.24841/fa.v31i1.570>

Karpowicz M, Ejsmont-Karabi Y, Rojas-Baez E, Pardo MJ, López C (2025) Rotifera of the Peruvian Andes: new records and insights. Diversity 17(3): 217. <https://doi.org/10.3390/d17030217>

Koste W (1988) Über die Rotatorien einiger Stillgewässer in der Umgebung der Biologischen Station Panguana im tropischen Regenwald in Peru. Amazoniana 10: 303–325.<https://repositorio.inpa.gov.br/handle/1/39885>

Murray J, Wailes GH (1913) Notes on the natural history of Bolivia and Peru. Scottish Oceanographical Laboratory, 45 pp.

Murrieta-Morey GA, Nájar J, Alcantara-Bocanegra F (2015) Producción experimental de rotíferos en bolsas de plástico utilizando harina de pescado como fuente de nutrientes. Folia Amazónica 24(2): 115–122.<https://doi.org/10.24841/fa.v24i2.67>

Ortega H, Rengifo B, Samanez I, Palma C (2006) Diversidad y el estado de conservación de cuerpos de agua Amazónicos en el nororiente del Perú. Revista Peruana de Biología 13: 189–193. <https://doi.org/10.15381/rpb.v13i3.2336>

* Osorio M, Romero S, Guabloche A, Alvariño-Flores L, Ayala-Sulca Y, Carrasco-Badajoz C, Castañeda L, Carrasco L, Iannacone JA (2022) Use of *Brachionus plicatilis* (Rotifera) to assess the quality of marine water in Callao Bay, Peru. Revista de Gestão Costeira Integrada 22: 103–116. [https://doi.org/10.5894/rgci-n44](https://doi.org/10.5894/rgci-n441)

Paredes C, Iannacone J, Alvariño L (2007) Biodiversidad de invertebrados de los humedales de Puerto Viejo, Lima, Perú. Neotropical Helminthology 1: 21–30.<https://doi.org/10.24039/rnh2007111149>

Richerson P, Widmer C, Kittel T (1977) The Limnology of Lake Titicaca (Peru-Bolivia), a Large, High Altitude Tropical Lake. Institute of Ecology, 14, 78pp.

Riofrío J, Samanez I, Carrasco F, Clavo M (2003) Caracterización limnológica de la laguna de Cashibococha (Ucayali-Perú) durante el año 2001. Revista Peruana de Biología 10: 183–194. <http://www.scielo.org.pe/scielo.php?script=sci_arttext&pid=S1727-99332003000200009>

Sánchez-Dávila PPA, Sotil G, Adabache-Ortiz A, Cueva D, Silva-Briano M (2021) Integrative Taxonomy of Two Peruvian Strains of *Brachionus Plicatilis* Complex with Potential in Aquaculture. Diversity 13: 671.<https://doi.org/10.3390/d13120671>

Samanez I (1988) Rotíferos Planctónicos de La Amazonía Peruana I. Departamento de Ucayali. Revista Peruana de Biología 3(1):1–4

Samanez I (1991) Rotíferos Planctónicos de La Amazonía Peruana II. Departamento de Loreto. Publicaciones del Museo de Historia Natural UNMSM 38: 1–4.

Samanez I, Riofrío C (1995) Composición de la fauna de rotíferos y su relación con las macrófitas acuáticas en una laguna fluvial, Ucayali. Publicaciones del Museo de Historia Natural UNMSM 50: 20–30.

Samanez I, Zambrano F (1995) Observaciones sobre la diversidad y algunas características ecológicas del plancton en el departamento de Madre de Dios. Perú. Publicaciones del Museo de Historia Natural UNMSM 51: 1–10.

*Schmarda LK (1859) Neue Turbellarien, Rotatorien und Anneliden. In: Schmarda LK, Engelmann W (Eds) Volume 1: Neue wirbellose thiere beobachtet und gesammelt auf einer reise um die erde 1853 bis 1857. Wilhelm Engelmann, Leipzig, 1–164.

* Schmidt SK, Darcy JL, Sommers P, Gunawan E, Knelman JE, Yager K (2017) Freeze–thaw revival of rotifers and algae in a desiccated, high-elevation (5500 meters) microbial mat, high Andes, Perú. Extremophiles 21: 573–580. <https://doi.org/10.1007/s00792-017-0926-2>

Thomasson K (1956) Reflections on Arctic and Alpine Lakes. Oikos 7(1):117–143.<https://doi.org/10.2307/3564988>

Toscano E, Severino R (2013) Brachionidae (Rotifera: Monogononta) de La Albufera El Paraíso y el reporte de *Brachionus Ibericus* en el Perú. Revista Peruana de Biología 20: 177–180.<https://doi.org/10.15381/rpb.v20i2.2683>

Zambrano F, Burger L (1992) Notas comparativas sobre la diversidad zooplanctónica de diez lagunas altoandinas en Huánuco, Perú. Boletín de Lima 19: 89–95.
